# Automatic evaluation-feedback system for automated social skills training

**DOI:** 10.1038/s41598-023-33703-0

**Published:** 2023-04-26

**Authors:** Takeshi Saga, Hiroki Tanaka, Yasuhiro Matsuda, Tsubasa Morimoto, Mitsuhiro Uratani, Kosuke Okazaki, Yuichiro Fujimoto, Satoshi Nakamura

**Affiliations:** 1 Graduate School of Science and Technology, Nara Institute of Science and Technology, Ikoma, 630-0192 Japan; 2 Osaka Psychiatric Medical Center, Hirakata, 573-0022 Japan; 3 Department of Psychiatry, Nara Medical University, Kashihara, 634-8521 Japan

**Keywords:** Computer science, Human behaviour, Rehabilitation

## Abstract

Social skills training (SST), which is a rehabilitation program for improving daily interpersonal communication, has been used for more than 40 years. Although such training’s demand is increasing, its accessibility is limited due to the lack of experienced trainers. To tackle this issue, automated SST systems have been studied for years. An evaluation-feedback pipeline of social skills is a crucial component of an SST system. Unfortunately, research that considers both the evaluation and feedback parts of automation remains insufficient. In this paper, we collected and analyzed the characteristics of a human–human SST dataset that consisted of 19 healthy controls, 15 schizophreniacs, 16 autism spectrum disorder (ASD) participants, and 276 sessions with score labels of six clinical measures. From our analysis of this dataset, we developed an automated SST evaluation-feedback system under the supervision of professional, experienced SST trainers. We identified their preferred or most acceptable feedback methods by running a user-study on the following conditions: with/without recorded video of the role-plays of users and different amounts of positive and corrective feedback. We confirmed a reasonable performance of our social-skill-score estimation models as our system’s evaluation part with a maximum Spearman’s correlation coefficient of 0.68. For the feedback part, our user-study concluded that people understood more about what aspects they need to improve by watching recorded videos of their own performance. In terms of the amount of feedback, participants most preferred a 2-positive/1-corrective format. Since the average amount of feedback preferred by the participants nearly equaled that from experienced trainers in human–human SSTs, our result suggests the practical future possibilities of an automated evaluation-feedback system that complements SSTs done by professional trainers.

## Introduction

For more than 40 years, social skills training (SST) has been used in clinical fields as a rehabilitation program to help improve clients’ communication and people skills. Although definitions of social skills vary widely, we follow Bellack’s approach, which can be divided into the following four factors: expressive behaviors (e.g., speech content), receptive behaviors (e.g., emotion recognition), interactive behaviors (e.g., response timing), and situational factors (e.g., an individual may be more assertive depending whether acquaintances or strangers are involved)^1,2^. A basic SST approach begins with a briefing about the session’s target skills, a vital step for sharing the target between clients and trainers to maximize the training effect. Then a trainer or another client role-plays good examples of the target skill being used. This enhances the training effect based on social learning theory, which fundamentally advocates learning by imitating^3^. Finally, the client acts in a role-play by himself, followed by feedback from the trainer on his performance.

Since clinicians worldwide have recognized SST’s effectiveness, its demand is increasing in clinical fields. Unfortunately, its accessibility is limited due to the lack of qualified SST trainers. Therefore, researchers are working on automated systems. To address job-interview performance, Hoque et al. developed a virtual agent system called MACH, which assesses social skills using nonverbal features^4^. Their system provides graphical summary feedback on a user’s performance following interactions in each job-interview session. For example, visual graphs correspond to such nonverbal features as smile intensity as well as speaking rate. Based on the ratings of human experts, students who participated in a week-long experiment improved their job-interview performances. Schneider et al. developed a public-speaking-training system called Presentation Trainer using body and voice features^5^. They tracked hand gestures, posture, voice volume, pauses, and fillers. Based on the tracked features, they offered immediate corrective feedback when an inappropriate behavior was recognized and interrupted a session if such behavior mistakes were excessively repeated. Their experimental result showed a significant reduction in the amount of such mistakes after five training sessions. Tanaka et al. implemented an automated SST system using a similar feature-based feedback approach^6^. They used audio, visual, and linguistic features to directly generate summary feedback. They identified the effectiveness of their system for both healthy control and ASD groups, a result that provides evidence for the applicability of an automated system to complement experienced trainers. The popularity of research on automatic social skill assessments continues to increase in presentation training, job interviews, and public speaking^7–9^.

Such works identified the effectiveness of an automated SST-like training system that simply uses automated features directly as training feedback. However, their investigation was limited to dyadic interactions (e.g., turn taking, backchannel) and the interactive effects between features. Although such assessments are crucial components for acquiring better social skills, implementing them remains technically challenging. To overcome this difficulty, Naim et al. estimated job-interview performances using a SVR machine learning model with multimodal features^10^. They employed nine crowd workers who rated 16 interview traits of videos on a 7-point Likert scale and used the averages as ground-truth job-interview performance scores for machine learning training. Their best overall performance achieved a correlation coefficient of 0.70.

**Figure 1:**
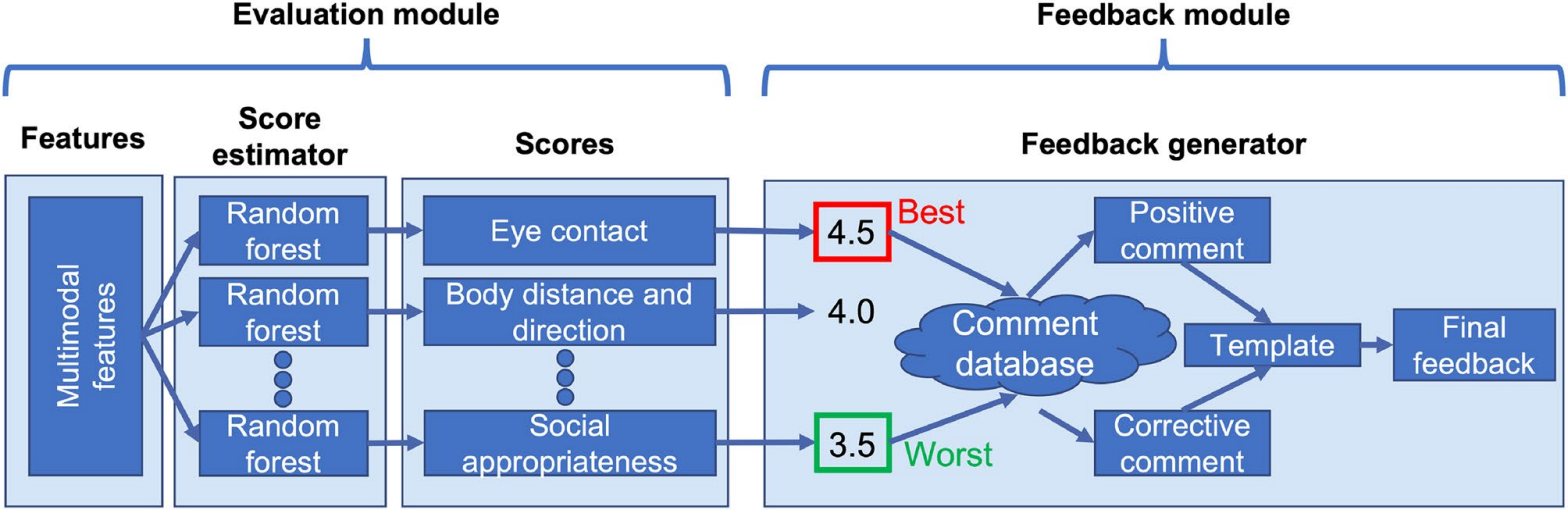
Schematic of our evaluation-feedback pipeline of social skill scores.

Similar to Naim’s approach, we estimated SST performances by using machine learning models. We implemented machine-learning-based social skill estimators for our automated SST system and examined its feasibility through user-studies. We compiled a human–human SST dataset of 226 sessions that included 19 healthy controls, 15 schizophreniacs, and 16 ASDs. To develop our feedback system, we first analyzed the feedback given by experienced trainers. According to self-efficacy theory, people who receive constructive feedback will be motivated to act, whereas those whose self-efficacy is lowered by feedback will become discouraged^11^. Therefore, how to give feedback is critical for SST outcomes; unfortunately, no concrete SST feedback method exists. For this reason, we developed an automatic system by first analyzing the feedback characteristics of experienced trainers. Based on this analysis result, we developed a social-skill-estimation model using both audio-visual and text features with machine learning techniques. Compared to previous visual feedback that directly corresponds to feature values, our system estimated social skills with multimodal machine learning models to consider complex skills based on the interaction among features. We also developed an automated evaluation-feedback pipeline with a summary feedback strategy, inspired by experienced SST trainers. At the core of our automated quantitative evaluation, we addressed complex evaluations by human trainers by establishing the following seven component scores: eye contact, body direction/distance, facial expression, vocal variation, clarity, fluency, and social appropriateness. Our system consists of two modules: an evaluation module for seven-component, social-skill-score estimation with machine learning models and an feedback module that generates positive/corrective responsive language based on the estimated seven component scores (Fig. 1). Using the system, we experimentally ran user-studies on several conditions. With the results of the estimation model performance and a series of user-studies, we show the feasibility of our automated evaluation-feedback pipeline for human-agent SST systems. This paper is an extended version of our previous publication^12,13^.

**Figure 2:**
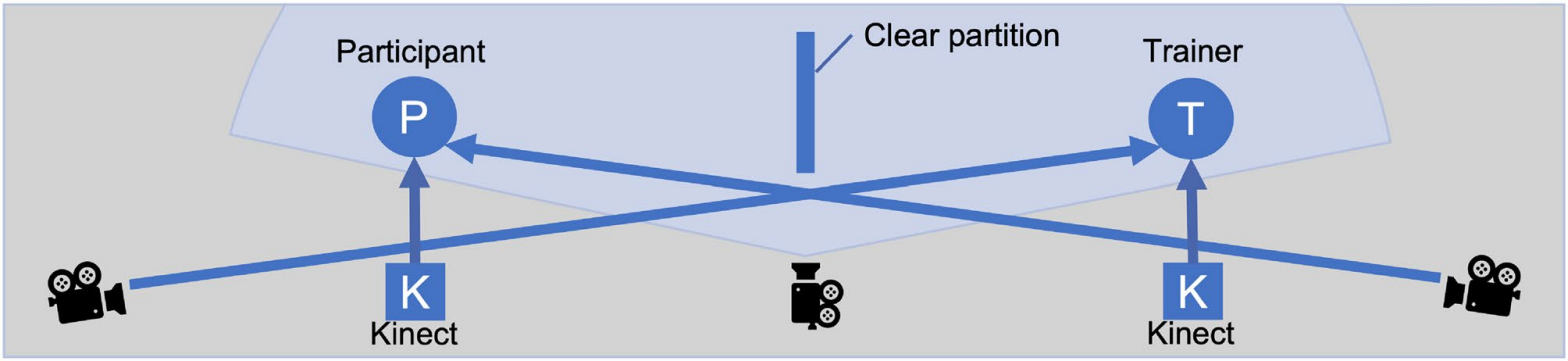
Top view of recording device map^12^.

**Table 1:** Dataset statistics: we used one-way ANOVA as the statistical test, where * indicates significant differences (p<0.05).

	Control	SZ	ASD	
Sex	M:10, F:9	M:7, F:8	M:10, F:6	
Assessment	Average (SD)	Average (SD)	Average (SD)	p value
Age	28.42 (4.06)	32.07 (9.13)	26.50 (5.85)	0.062
SRS	64.68 (30.08)	n.a.	73.00 (32.87)	0.440
FEIT	14.89 (2.51)	14.27 (2.52)	14.75 (3.00)	0.785
KISS-18	60.95 (13.86)	59.07 (9.14)	45.81 (9.34)	0.001*
Singelis	137.00 (17.58)	140.67 (13.87)	131.63 (15.39)	0.287
PANSS	n.a.	− 1.18 (1.64)	n.a.	n.a.
BACS: verbal memory and learning	n.a.	− 1.04 (1.24)	0.06 (0.85)	0.002*
BACS: working memory	n.a.	− 2.35 (1.65)	0.02 (0.78)	0.010*
BACS: motor function	n.a.	− 0.63 (1.85)	− 1.25 (1.35)	0.023*
BACS: verbal fluency	n.a.	− 1.42 (1.39)	0.02 (1.27)	0.193
BACS: attention and information processing speed	n.a.	− 0.36 (1.65)	− 1.20 (0.99)	0.474
BACS: processing function	n.a.	67.07 (12.99)	0.23 (1.44)	0.150

## Methods

### Dataset

We used a human–human SST dataset collected in our previous research^12^. It includes data from 49 participants with the following characteristics: 15 ASDs, 15 schizophreniacs (SZ), and 19 controls. We collected these psychiatric or developmental difficulty groups to develop our automated SST system and to investigate their interaction differences. We included these groups since the main SST clients in clinical facilities are ASDs and SZs in Japan. We combined data from the control group and the clinical groups to train the social-skill-estimation models to maximize the training dataset’s size because machine learning methods generally require a lot of data for better estimation stability. Furthermore, this approach makes it possible to extend our system target to the general population. For example, our social skills training system will probably be useful for job-hunting students or people who struggle to communicate with co-workers. The participation of clinical groups can also absorb the skewed distribution of social skill scores since the control group’s scores were biased toward the better side. In contrast, the scores of the clinical groups were generally on the worse side. Therefore, mixing the data groups provides less biased training of the machine learning models. To accelerate this balancing operation, we combined the data from ASD and SZ people. We combined those symptomatic groups since there are reports that people with ASD and SZ share several symptomatically similar characteristics, such as a flat or a blunted affect (e.g., reduced eye contact) or alogia (e.g., impoverished speech)^14–16^. At the same time, such two-group data collection can also be used to generate personalized, symptom-specific feedback. Bellack et al. recommended using shorter, more precise feedback for those with schizophrenia who are also rather likely to suffer from hallucinations^1^. Since hearing voices is distracting and might derail SST sessions, trainers struggle to identify a problem to focus on. For ASD participants, feedback might be disengaging if trainers frequently mention exaggerated gestures and facial expressions since they are major symptomatic characteristics. Furthermore, research suggests some social deficits (e.g., visual social attention related to facial image perception in ASD) are related to abnormal brain activities^17^. Since improving these deficits is complicated, SST should focus on other, more recoverable skills. For this reason, the data in an SST setting with the identical environment will be helpful for the future developments of a personalized SST system. Although we plan to examine the effectiveness of our system with clinical groups, for ethical and safety reasons we first target healthy controls until we prove the effectiveness of our approach. Based on these safety assurances, we will extend our target to them.

Our dataset includes SST sessions of four role-play tasks for each participant based on Bellack’s definitions^1^ of the four basic social skills: LISTEN: paying attention to others, TELL: conveying positive feelings, ASK: requesting from others, DECLINE: refusing a request. Some participants repeated SST sessions with the same SST task two or three times for further improvements. The length of each session ranged from 30 s to 2 min. The dataset includes 168 sessions (LISTEN: 43, TELL: 34, ASK: 46, DECLINE: 45). Since it is better to have a larger dataset to boost the robustness of the machine learning training, we combined additional human-agent interaction data with a similar recording protocol^18^. Therefore, our extended dataset for the machine learning models resulted in 276 sessions (LISTEN: 70, TELL: 61, ASK: 73, and DECLINE: 72). We used the core dataset with 168 sessions for the feedback content analysis and the extended dataset with 276 sessions for the machine learning training.

We recruited the control participants from a temporary recruitment agency and the clinical groups from those with SST backgrounds from the Nara Medical University and Heartland Shigisan Hospitals. All the participants underwent a medical interview prior to the examination to confirm the presence/absence of eye diseases as well as a history of psychiatric outpatient/inpatient treatment. The interviews were conducted by examiners or experienced psychiatrists. Participants with eye diseases were excluded. Those in the control group with a history of psychiatric outpatient/inpatient treatment were also excluded. For the clinical participants, we excluded those who scored less than 70 on the third edition of the Wechsler Adult Intelligence Scale (WAIS-III) IQ test^19^. We set the sample size by taking the balance of the generality of the results and the practical workload for the data collection. The data collection period ranged from January 2020 to January 2021.

We tested each participant using the following clinical assessments for further analysis: the Facial Emotion Identification Test (FEIT)^20^, Kikuchi’s Scale of Social Skills: 18 items (KiSS-18)^21^, Singelis’ Independent-Interdependent Self-construal Scale (Singelis)^22^, the second edition of the Social Responsiveness Scale (SRS-2)^23^, and the Japanese version of the Brief Assessment of Cognition in Schizophrenia (BACS-J)^24,25^. FEIT, which assesses the emotional perception of facial emotions, includes facial images in a grayscale of 19 different people with one of six emotions: happiness, sadness, anger, surprise, fear, or shame. We included this assessment because people with ASD and SZ struggle to recognize emotions in facial images^26,27^. KiSS-18, which measures social skill levels, is composed of 18 questions based on six categories defined by Goldstein^28^. This metric comprehensively measures social skills. Singelis, which consists of 30 questions on a 7-point rating scale, measures how people view themselves in relation to others. SRS-2, composed of 65 questions, is an evaluation metric of the severity of social impairment. Although it was initially designed to assess people with potential ASD, it can also differentiate among various mental difficulties. Its effectiveness has been investigated with both clinical and healthy populations^23^.

We used BACS-J to quantize the impaired aspects of cognition for the SZ and ASD groups. BACS-J is the Japanese version of BACKS, which assesses the aspects of cognition found to be most impaired and most strongly correlated with outcomes in SZ patients. Since not only the SZ group but also the ASD group include cognitive impairments, we also applied BACS-J to the latter.

In addition, we used the Autism Disorder Observation Schedule, 2nd Edition (ADOS-2) for the ASD group and the Positive and Negative Syndrome Scale (PANSS) for the SZ group^29,30^. ADOS-2 is a semi-structured assessment that includes several play-based activities for collecting information related to communication, social interactions, and restricted and repetitive behaviors associated with ASD. PANSS, a typological and dimensional assessment instrument for SZs, is composed of 30 standardized items and provides a balanced representation of positive and negative symptoms. Table 1 shows the detailed statistical characteristics of this dataset.

Figure 2 shows a device map of the data recording. Facial video and audio were recorded with video cameras located diagonally toward the participant and the trainer. We also set an additional camera in the center to record the situation’s overview and the experimental condition. Body movements were captured using a pretrained pose estimator with two Azure Kinects: one on the trainer’s side and another on the participant’s side^31^. Even though the meaning of gestures in communication is essential, automatically quantizing them is difficult. In contrast, communicative distance in social interaction is as crucial as gestures, simplifying their automatic quantization. Although we could capture every body movement if we placed a Kinect diagonally, like a video camera in Fig. 2, this arrangement complicates the communicative distance calculation. Therefore, for this study, we put the Kinects on the sides of the participant and the trainer to focus on the distance calculation. We chose to avoid placing standing position marks for the participants to stimulate a natural communicative distance between the participants and the trainers in the SST sessions. Our annotator transcribed the language of the participants and the trainers from the video and audio files.

**Table 2:** Input features used for our automated evaluation models.

Feature name	Explanation
BERT_self_self_sent	Sentence-level intra-person BERT-based seq-similarity
BERT_self_self_cont	Content-word-level intra-person BERT-based seq-similarity
BERT_self_inter_sent	Sentence-level inter-person BERT-based seq-similarity
BERT_self_inter_cont	Content-word-level inter-person BERT-based seq-similarity
Num_content	Number of content words
Thanks_flag	Binary flag for presence of appreciative language
Sorry_flag	Binary flag for presence of apologetic language
Explicit_refuse_flag	Binary flag for presence of explicit refusal language
Num_backchannel	Number of backchannels
Init_cue_flag	Binary flag for presence of initialization cue words
WPM	Words per minute
Ave_voice_int	Average voice intensity
CV_voice_f0	Coefficient of variation of F0
Smile_freq	Percentage of smile frequency ranging from 0.0 to 1.0
Head_mean	Mean value of head poses (treated x, y, z values in the same axis)
Head_CV	Coefficient of variation of head poses
Num_nod	Number of nods
Mutual_smile	Percentage of mutual smile frequency ranging from 0.0 to 1.0
AU(XX)_r_(mean, CV)	Mean intensity and coefficient of variation of facial action units 01, 02, 04, 06, 07, 09, 10, 12, 15, 17, 20, 23, 25, 26, 45, ranging from 0.0 to 1.0
Gesture_all_CV	Coefficient of variation of all body joints
Gesture_upper_CV	Coefficient of variation of upper body joints
Gesture_arm_CV	Coefficient of variation of arm body joints

The most crucial score labels for automating social skills evaluations are subjective scores annotated by experienced SST trainers. Two different trainers watched role-play videos to evaluate the participants’ scores of the following seven components: eye contact, body direction/distance, facial expression, voice variation, clarity, fluency, and social appropriateness for each task. We utilized evaluation components from the revised version of the Japanese role-play test, which is a well-validated evaluation method for SST^32^. Although there are several other evaluation metrics, such as Assessment of Interpersonal Problem-Solving Skills (AIPSS) or Social Performance Rating Scale (SPRS), we chose the Japanese role-play test since it validates simple components and is written in Japanese^33,34^. Since the required skills depend on each situation, social appropriateness differs by each SST task. The LISTEN task, which determines whether the participants paid attention to the interlocutor, includes nodding, back-channels, and empathetic behaviors. For the TELL task, social appropriateness is about expressing attention to the interlocutor’s responses and the suitability of their speech contents. For the ASK task, social appropriateness assesses how thoroughly they explained their request, including what kind of help they need. It also includes whether they listened to the interlocutor. For the DECLINE task, social appropriateness is concerned with whether they expressed contriteness and appropriate reasons for a refusal. It also includes whether they proposed other options that they can provide to satisfy the interlocutor’s request, which is an essential act for the situation. Similarly, two other trainers evaluated the trainer’s actions with the following four components: appropriateness of positive feedback, suggestions for improvement, a good example of appropriate nonverbal communication, and a good example of appropriate verbal communication. Both evaluations were done on a 5-point Likert scale, from 1 (worst) to 5 (best). For instance, for eye contact, the evaluators give 5 points if the user’s eye contact is natural and occurs with appropriate frequency. Conversely, they give 1 point if the user has no eye contact, which the user might give the interlocutor a negative impression. We calculated Cohen’s quadratic kappa scores to validate the evaluation scores’ reliability. The reliability was confirmed with a kappa score of 0.84 across every subjective evaluation. We found no significant differences in the kappa scores among different SST tasks. On the other hand, we found significantly lower kappa scores for the healthy controls when comparing them with the SZs (0.78 and 0.83, respectively) and with the ASDs (0.78 and 0.88, respectively) with a Chi-square Test of Independence based on the frequency matrices of score differences between two annotators (p< 0.05). This result suggests that the variability of the social skill levels of the healthy controls was more diverse than in the other groups. Similarly, the kappa scores for the participants’ evaluations were lower than those of the trainers. However, we found no significant difference between the SZs and ASDs (p = 0.11). This SZs-ASDs closeness might come from the symptomatic similarity of SZs and ASDs^14–16,35^.

We explained the details of the experiments to all the participants and received their informed consent concerning the data collection, data usage, online open-access publication of analysis results, excluding such personal information as facial images or names (the individual in Fig. 4 is one of the authors). Following our institutional guidelines, participants were paid when they completed the experiments. Our data collection process was authorized by the institutional review boards of both the Nara Institute of Science and Technology and the Nara Medical University. This study was conducted according to the guidelines laid down in the Helsinki Declaration (2013 version).

### Feedback content analysis

Before developing an estimation model, we first investigated the feedback contents in human–human SSTs by experienced trainers to improve our input feature set. Since professional feedback doesn’t have explicit categories, we grouped it into types by manually checking the transcriptions and separately counted the amount of positive and corrective feedback for every type. Based on the frequency distribution of the feedback types, we investigated the tendency of the feedback that was given to determine which social skills are carefully monitored and frequently mentioned. Using this separate amount of positive and corrective feedback, we determined its optimal balance during SST sessions toward system automation. Based on the analysis result, we constructed a multimodal feature set.

### Social skill estimation

Table 2 shows a complete list of the input features used for our automated evaluation models. With the feedback tendency of experienced trainers from the previous analysis, we developed a multimodal feature set for a further automation process (see our previous paper^13^ for implementation details) We measured the naturalness of the connections of consecutive words with sequential similarity^36,37^, which uses Bidirectional Encoder Representations from Transformers (BERT), a neural network-based language model, to calculate the vectorized word or sentence representations^38^. We obtained the sequential similarity by taking the average cosine similarity between consecutive words and calculating it at the sentence and content word levels for the participant utterances. We extended it to capture the appropriateness of the participant-trainer interactive conversations. Similar to those for participant utterances, we calculated the sentence level and content word level features for the concatenated, time-aligned participant-trainer utterances. Although previous researchers used a wide variety of audio features^4,6,10^, unfortunately, we could not capture any other features since the audio quality was poor for the following reasons. First, since we used the default microphones of video cameras, the distances between the microphone and the speaker’s mouth were different. Second, the audio included such loud environmental noises as sounds from air conditioners. We also calculated several visual features, including facial and body features. As the target score of our estimation models, we chose seven-component subjective scores, which were annotated by the experienced trainers introduced in the dataset section.

**Figure 3:**
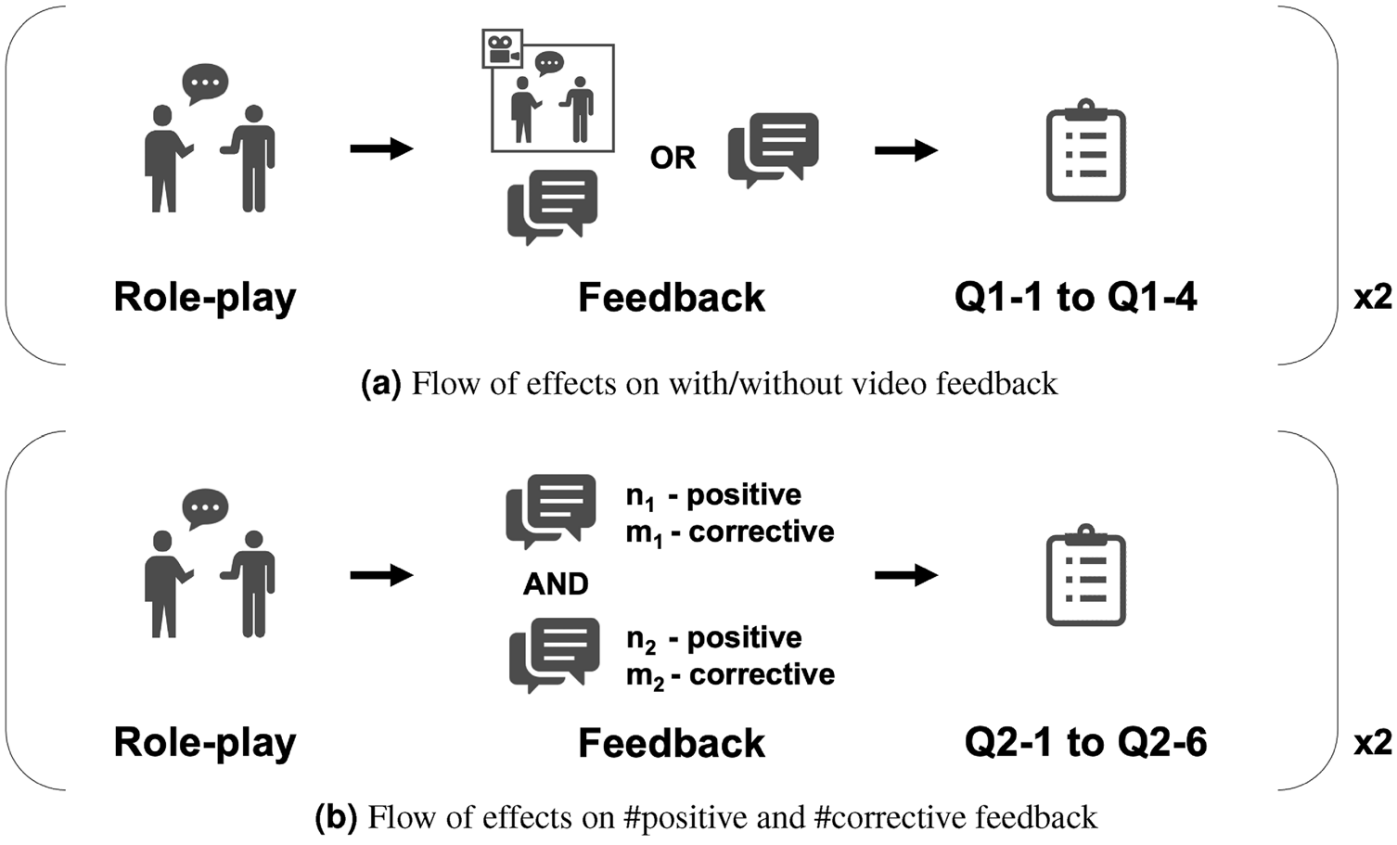
Flow of user-studies on feedback conditions.

**Figure 4:**
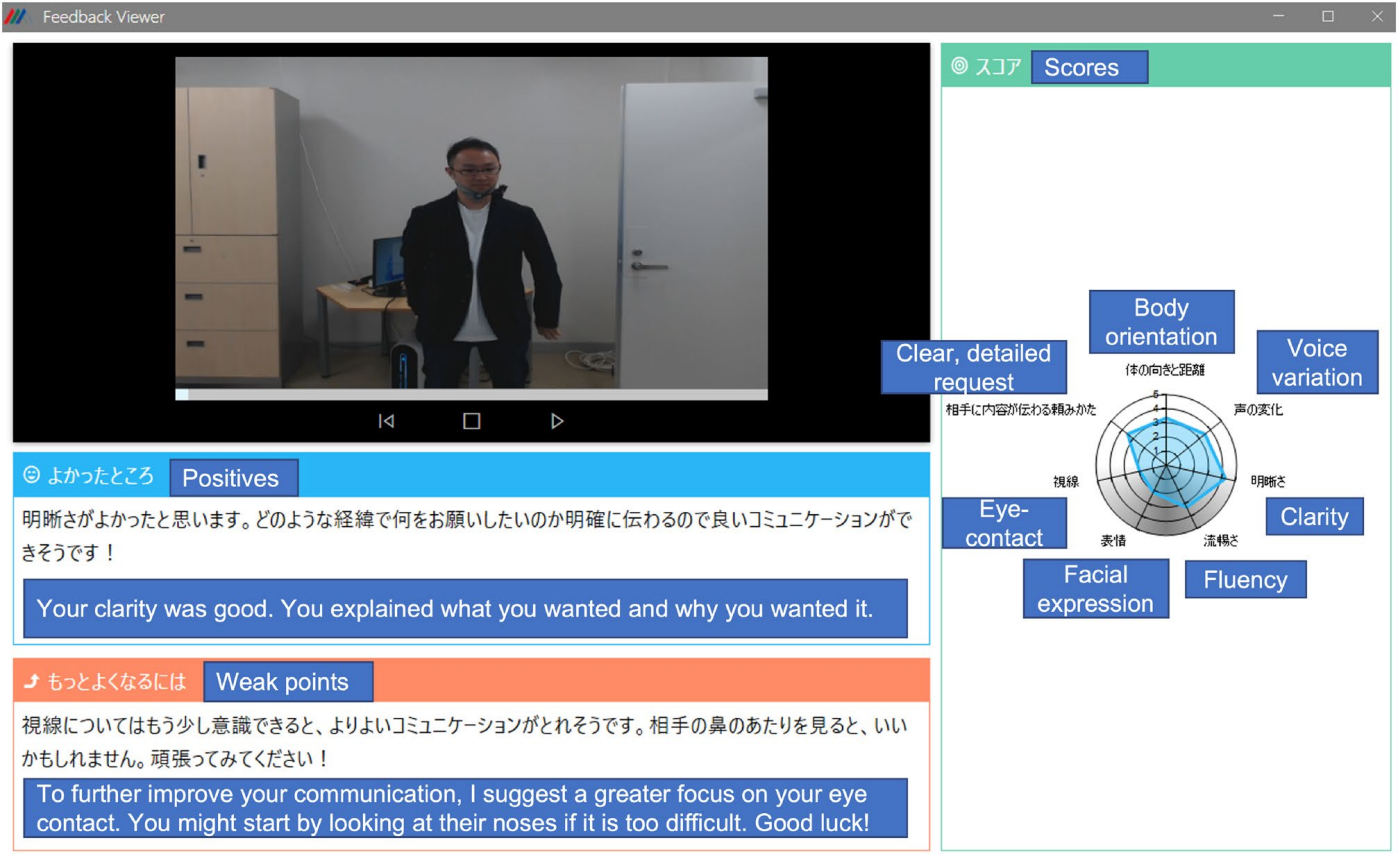
Feedback view of our SST system: from left-top to left-bottom: recorded video, positive comment, corrective comment, radar chart of predicted skill scores.

**Figure 5:**
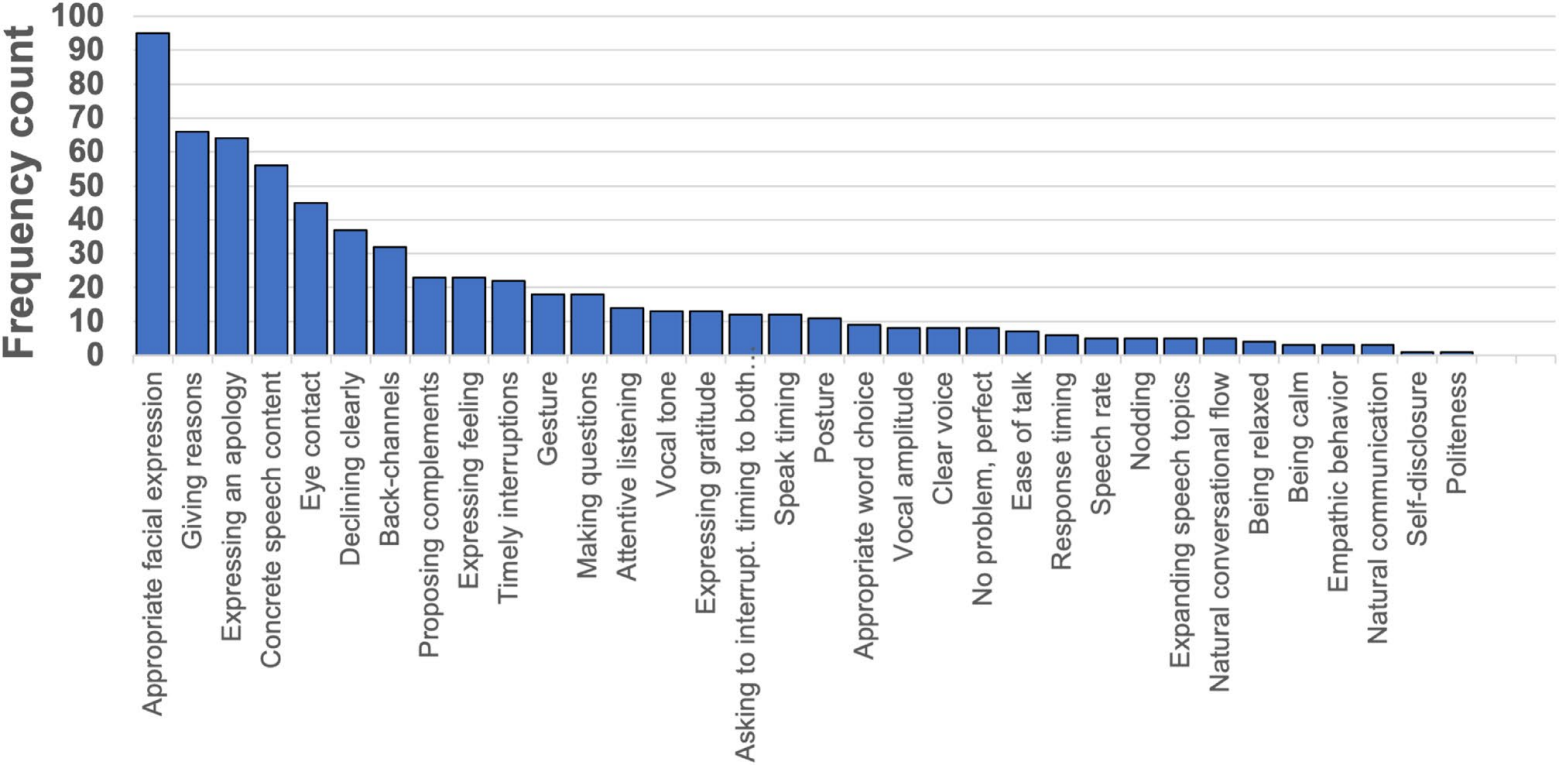
Frequency count of feedback by experienced trainers.

**Table 3:** Top-10 frequent feedback.

Feedback types	#Count	#Positive	#Corrective
Appropriate facial expressions	95	64	31
Giving reasons	66	55	11
Expressing apologies	64	41	23
Concrete speech content	56	48	8
Eye contact	45	38	7
Declining clearly	37	35	2
Back-channels	32	24	8
Offering compliments	23	18	5
Expressing feelings	23	21	2
Timely interruptions	22	19	3

**Table 4:** Model comparison: * denotes significant correlation in no-correlation test (p<0.05).

Model	LISTEN		TELL		ASK		DECLINE	
	$R^2$	Correl.	$R^2$	Correl.	$R^2$	Correl.	$R^2$	Correl.
PLS	− 0.71	0.20*	− 0.78	0.10*	− 0.42	0.32*	− 0.24	0.47*
SVR_rbf	0.15	0.42*	0.13	0.37*	0.13	0.42*	0.09	0.38*
Random forest	0.13	0.38*	0.16	0.39*	0.2	0.45*	0.16	0.42*
Neural network	− 1.76	0.31*	− 2.27	0.34*	− 1.14	0.31*	− 1.76	0.31*
VAE for regression	− 1.1	0.33*	− 1.79	0.36*	− 0.86	0.44*	− 0.88	0.49*

**Table 5:** Result of social-skill-score estimation: * denotes significant correlation in no-correlation test (p<0.05).

Task	Label	$R^2$	RMSE	Correl.
LISTEN	Eye contact	0.01	0.70	0.14
	Body direction and distance	0.00	0.71	0.22*
	Facial expressions	0.04	1.18	0.26*
	Vocal variation	0.04	1.23	0.26*
	Clarity	− 0.02	1.12	0.05
	Fluency	0.13	1.01	0.38*
	Social appropriateness	0.18	1.00	0.47*
TELL	Eye contact	0.11	0.57	0.29*
	Body direction and distance	0.19	0.65	0.45*
	Facial expressions	0.15	1.26	0.37*
	Vocal variation	0.12	1.05	0.31*
	Clarity	0.01	1.08	0.13
	Fluency	0.17	0.90	0.33*
	Social appropriateness	0.04	1.09	0.04
ASK	Eye contact	0.06	0.82	0.22
	Body direction/distance	0.11	0.51	0.33*
	Facial expressions	0.03	1.27	0.21
	Vocal variation	0.09	1.16	0.24*
	Clarity	0.40	0.79	0.68*
	Fluency	0.03	1.07	0.24*
	Social appropriateness	0.34	0.93	0.63*
DECLINE	Eye contact	− 0.06	1.05	0.04
	Body direction/distance	0.14	0.74	0.39*
	Facial expressions	0.03	1.44	0.19
	Vocal variation	0.07	1.53	0.28*
	Clarity	0.07	1.66	0.33*
	Fluency	0.12	1.33	0.38*
	Social appropriateness	0.15	1.26	0.36*

**Table 6:** $R^2$ of ablation study on modalities.

Feature modality	LISTEN	TELL	ASK	DECLINE
V+A+L	0.13	0.16	0.20	0.16
A+L	0.13	0.04	0.10	0.20
V+L	0.11	0.17	0.21	0.16
V+A	0.14	0.12	0.17	0.11

**Figure 6:**
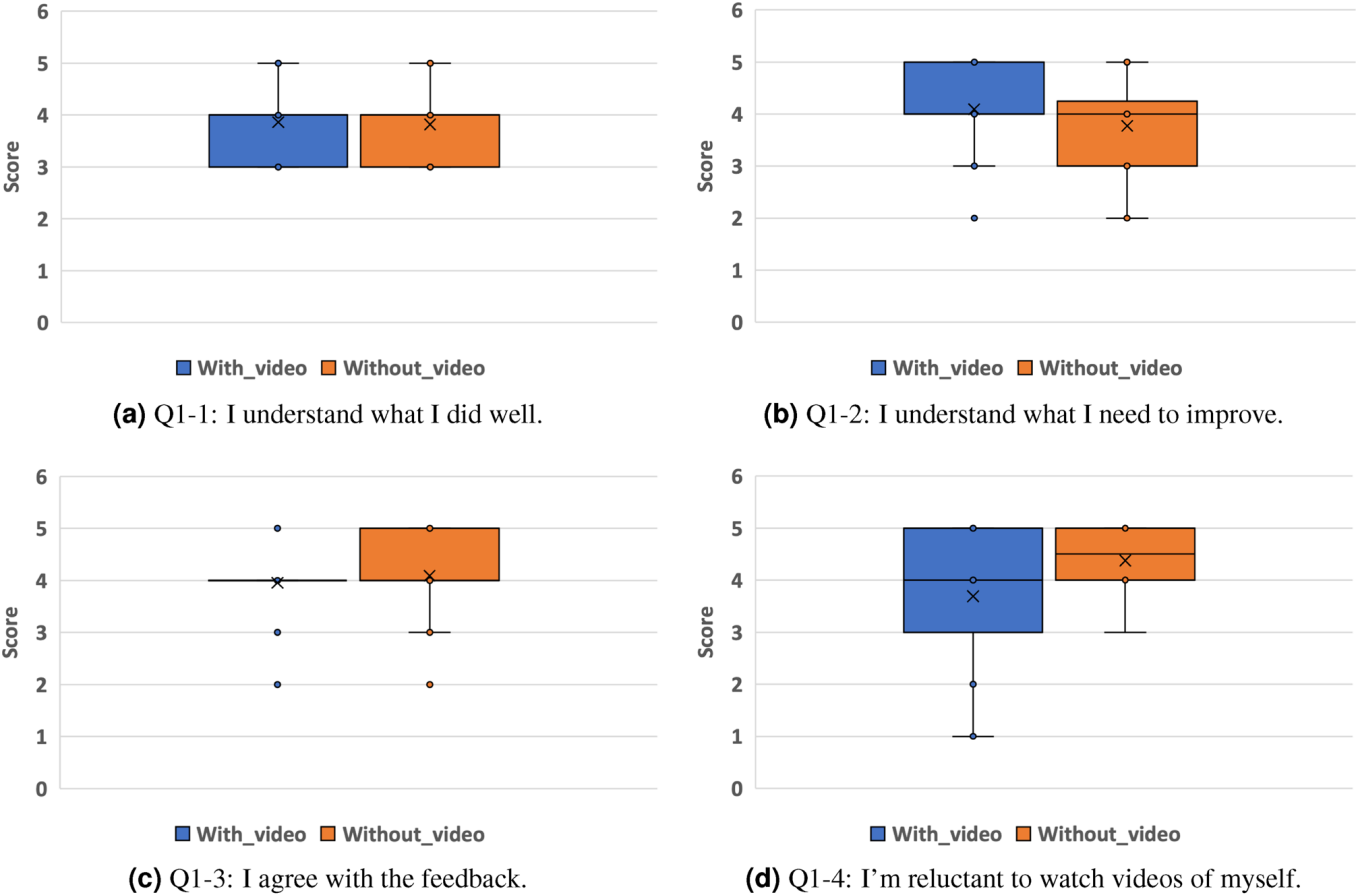
Questionnaire results concerning video feedback.

**Table 7:** Questionnaire summaries about user feedback preferences.

Question	#Positive			#Corrective		#Sum		
	0	1	2	0	1	1	2	3
Q2-1: I understand what I did well	4	12	26	11	31	7	17	18
Q2-2: I understand what I need to improve	8	13	19	3	37	8	16	16
Q2-3: The contents were easy to understand	8	14	20	5	37	8	19	15
Q2-4: I’m satisfied with the feedback	6	12	24	6	36	6	18	18
Q2-5: What feedback length do you prefer	6	18	16	5	35	8	19	13
Q2-6: What kind of feedback do you generally prefer	6	14	22	6	36	7	18	17

To investigate the best model for our problem, we started with a prediction model comparison with partial least square (PLS) regression, SVR, random forest, dense neural network, and a variational autoencoder (VAE) for the regression model^39^. We included SVR because it outperformed LASSO regression in Naim’s study. Random forest is known for its over-fit robustness for small-size datasets. Furthermore, since a recent study proved its stability in learning theoretically, we adopted it for our study^40^. Additionally, we added PLS regression and *VAE for regression* model, which can compress sparse information (e.g., our multimodal features) to a lower dimensional space. We also experimentally tried to construct an original neural network since recent neural network methods have shown promising results^41,42^. Since PLS, SVR, and random forest cannot estimate multiple scores simultaneously, we trained separate models for each component skill score. In contrast, since neural networks can simultaneously estimate various scores, we trained them in a simultaneous multi-output format. This approach should be more suitable for social skill estimation since it can consider inter-score interactions, which is impossible with a single-output format. As an additional experiment with the best performance model, we ran an abrasion study on feature modalities to investigate its effects on each fundamental social skill.

Since each person has a unique use style for social skills with a wide variety, we must consider individuality effects to prevent data leakage in machine learning. For instance, one participant expressed gratitude with odd hand gestures and a smile; another used a high tone of voice. Although machine learning methods can estimate ambiguous values, they tend to over-fit training samples when the dataset size is small and include a participant with a characteristic communication style. Therefore, to prevent this problem, we employed leave-one-participant-out cross-validation in our training. We first selected one participant as test data, followed by one participant selection as validation data. We used the remaining data as the training set. Regarding SST task-specific characteristics, since each SST task requires different skills, we used only the corresponding task’s data for training the PLS, the SVR, and the random forest. In contrast, since dense neural networks and *VAE for regression* require more data than the others, we used every SST session of every SST task for training, validation, and testing. For the PLS regression, we set the number of components to 7, with feature scaling normalization. For the SVR, we set the kernel type to the radial basis function with scaled gamma with $1/(n_{features}*X_{var})$ where $n_{features}$ denotes the number of input features and $X_{var}$ denotes their variance. The polynomial degree was 3, and regularization parameter *C* was 1.0. For the random forest, we set the number of estimators to 100, the maximum number of selected input features to the squared root of the number of input features, the maximum depth of each node to 5, and a function to measure the quality of the split to the mean squared error. We constructed five blocks for the dense neural network (fully connected layers with 30 nodes, 1-dimensional batch normalization, ReLU activation function) followed by outputting a fully connected layer. For the dense neural network training, we used the following setup: Adam with a learning rate of 0.001, beta 1 to 0.9, beta 2 to 0.999, a batch size to 10, a loss function to the mean squared error, and maximum steps to 300 with the early stopping of 5-step patience. For the *VAE for regression* model, we used the original network specifications with minor modifications of the hidden and latent dimensions, which we set to 10 and 5^39^.

### User-study on feedback methods of automated SST system

Since professional SST trainers evaluate their clients’ social skills based on their experience and partially-subjective evaluations of their clients’ attitudes, automation is technically challenging due to ambiguous subjectivity. Therefore, we made a component-score-based evaluation pipeline for our system (Fig. 1). We generated positive- and corrective-feedback sentences based on the estimated scores of pre-defined templates and prepared positive and corrective feedback templates. A positive template has placeholders for component names and corresponding advantages for skill use. For a corrective template, we set placeholders for component names and corresponding tips for improvement. Although there are several feedback theories, such as sandwich feedback^43^, we chose the simplest, the best, and the worst scores as our system’s strategy. Using the predicted scores, we constructed feedback with the best- and worst-scored components. As Bellack et al. suggested, we placed the positive feedback immediately after the role-play followed by corrective feedback^1^. For example, the following is translated feedback for the ASK task under a condition with the best score for clarity and the worst score for facial expression where brackets indicate placeholders: *Your [NAME_POSITIVE:clarity] was fantastic. Since you can [ADVANTAGE:directly say what you want to ask and give a reason], your communication is good. It will improve when you pay more attention to [NAME_CORRECTIVE:facial expressions]. It might also be effective to [TIPS:ask honestly without too much smiling]. Keep it up!* We subjectively confirmed the quality through a preliminary experiment with our laboratory members for improvement under the supervision of experienced trainers. We modified the lengths and feedback templates of each feedback through this examination. With this evaluation-feedback pipeline for an SST system, we ran user-studies to investigate the best strategy for our automated feedback.

The first test addressed the effect of the recorded videos. Figure 3a shows the overall flow of our experiment. First, a participant did an SST role-play session with the system. Second, the system automatically generated a feedback view with or without recorded video. Third, the participant completed a questionnaire about the ease of understanding and her preferences by selecting one from the following choices: *“I understood well”, “I understood but not completely”, “I didn’t understand very well”, and “I didn’t understand at all”*. We used the following questions (Q1-1 to Q1-4, translated from Japanese): *“I understand what I did well.” “I understand what I need to improve”. “I agree with the feedback content”. “I’m reluctant to watch videos of myself”.*. The participant repeats this loop with the opposite video condition (with or without). We randomized the order of the video conditions to eliminate any order effects. In addition, we randomly selected a target training task for each role-play from the following four tasks: listening to others (LISTEN), conveying positive feelings (TELL), requesting from others (ASK), and declining a request (DECLINE).

The second test, for which we used a comparison test, focused on the amount of positive and corrective feedback. Figure 3b shows the overall flow of this experiment. First, a participant does an SST role-play session with the system. Second, an experimenter randomly shows side by side on a screen two feedback views (A and B) with a different number of positive and negative settings. Figure 4 shows an example of the feedback view, which includes a recorded video, a radar chart of the estimated scores, and positive and corrective feedback comments. Third, the participant fills out the following questionnaire about ease of understanding and preferences by selecting A or B for each question/statement: *“Q2-1: I understand what I did well”. “Q2-2: I understand what I need to improve”. “Q2-3: The contents were easy to understand”. “Q2-4: I am satisfied with the feedback contents”. “Q2-5: What feedback length do you prefer?” “Q2-6: What kind of feedback do you generally prefer?”*. For the experiment, we used a feedback strategy with *n* positive and *m* corrective feedback, where *n* and *m* denote the amount of positive and corrective feedback. Based on clinical experience in human–human SST, we set the range of *n* from 0 to 2 for positive feedback and from 0 to 1 for *m* for the corrective type. This decision also follows a suggestion by Bellack et al., which concluded that positive feedback should always precede corrective or negative feedback^1^. Although we’ve tested conditions with more items that could provide a more comprehensive comparison, we received comments about the difficulty of concentrating on lengthy feedback. Therefore, by balancing workload and comprehensiveness, we limited the amount of feedback. For each participant, we ran two loops of the role-play/questionnaire for each test. Similar to the first experiment, we randomly selected one target training task out of the four types for each role-play. 21 participants joined the experiment; we obtained 42 samples. To confirm its appropriateness, we also manually counted the amount of positive and corrective feedback separately for feedback by experienced trainers in the human–human SST dataset based on the manual feedback classification explained in the method section of the *Feedback content analysis*.

## Results

### Feedback content analysis

Figure 5 shows the distribution of feedback by experienced trainers, where each bar denotes a different type. We confirmed 37 types based on their feedback. The top-10 frequent feedback occupied 71% of all 37 types.

Table 3 shows detailed statistics of the top-10 feedback. The amount of positive feedback always exceeded the corrective type. Four feedback types (*Gave reasons, Concrete speech content, Proposed alternatives, and Checked whether they had time to talk*) addressed speech content: linguistic features. In contrast, the remaining six feedback types concerned both verbal and nonverbal skills: para-linguistic features. We constructed input features based on these results. For example, we measured *Appropriate facial expressions* by using the intensity values of the facial action units of OpenFace as input to the evaluation models. We measured *Concrete speech content* by counting the content words (e.g., nouns, verbs, adjectives, nominal adjectives, adverbs) of each user’s utterances. To capture *Expressing apologizing* and *Declining clearly*, we prepared a set of keywords to judge whether they satisfied the requirements.

### Social skill estimation

Table 4 shows the model comparison results for the social-skill-score estimation, where *Correl.* indicates the Spearman’s correlation coefficient, and values with * indicate a significant correlation in the no-correlation test. To compare the model performances at the abstract level, the table shows the component-merged task scores ($R^2$ and Spearman’s correlation coefficient). We confirmed that every model achieved significantly correlated predictions for all the tasks, although there are differences in the levels of the statistics. In terms of the $R^2$ scores, since the random forest model showed the best or the most competitive performance across the tasks, we investigated it in more detail.

Table 5 shows the result of the social-skill-score estimation, where *Correl.* denotes Spearman’s correlation between the ground truth and the estimated scores. Scores with * in the *the Correl.* column showed a significant correlation in the no-correlation test (p < 0.05). For each SST task, we confirmed that five of the seven component models were significantly correlated, especially *Body direction and distance, Vocal variation*, and *Fluency*, all of which showed significance for every task. The maximum correlation value was 0.63 for the *Social appropriateness* of the ASK task.

Table 6 shows the $R^2$ scores in the modality ablation study with the random forest model, where *V* denotes the visual modality, *A* denotes the audio modality, and *L* denotes the linguistic text modality. By dropping the visual modality, the LISTEN performance decreased by 0.12 points, and the TELL performance decreased by 0.10 points. For other modalities and tasks, we didn’t confirm any apparent differences.

### User-study on feedback methods

Figure 6 shows the result box plot of the questionnaires on video feedback. We found no significant differences between the with/without video feedback conditions for every question with a Mann–Whitney U test (p < 0.05).

Table 7 shows the result of the user preferences on different feedback lengths. Their preferences were unequally distributed in every question based on a test for the goodness of the fit to equal distribution (p < 0.05). Generally, our participants preferred more feedback over less input for positive feedback. Similarly, they preferred corrective feedback rather than a condition without any corrective feedback. In terms of the total amount of feedback, more than two feedback contents were preferred over just one.

## Discussion

In this paper, we collected a human–human SST dataset comprised of healthy controls, schizophreniacs, and ASDs. By analyzing the characteristics of the feedback from experienced trainers in the dataset, we identified several frequently given types. Based on this finding, we developed an automated evaluation-feedback pipeline. Using this system, we found the best quantity balance of 2-positive/1-corrective feedback from a user-study and described the feasibility of an automated evaluation-feedback system for human-agent SSTs.

Since the top-10 frequent feedback occupied 71% of all the 37 types, this indicates the importance of such skills in the SST feedback phase. However, since the direct implementation of an automatic feedback system of these skills is technically challenging, we must modify some of them for an automated system. For example, measuring *Concrete speech content* is difficult since no obvious method exists. We captured such content by counting the number of content words. Another area for improvement is the diversity of the feedback types. Although we confirmed 37 different types of feedback within our SST dataset, implementing every kind is nearly impossible. Furthermore, even though experienced trainers confirmed its validity, the first author annotated and grouped these types. Therefore, it is potentially subjective and might differ from one annotator to the next. In this study, to absorb this limitation, we converted the automatic features inspired by the feedback analysis to the 7-component scores from the SST-role-play-test^32^. However, its reliability must be verified by calculating the inter-rater agreements in future work. We should remember that human trainers’ evaluation processes in clinical SSTs are conceptually different from the SST-role-play-test since the former is adaptive to the client’s needs, whereas the latter uses pre-defined situational settings. Automating this adaptive SST could be an interesting technical challenge, whose possibility is rising with the emergence of epic dialogue models, such as the family of Generative Pretrained Transformers (GPTs), e.g., InstructGPT or its chat-specialized version chatGPT^44^.

We compared the models on social skill estimation in Table 4, and they generally achieved correlation coefficients of around 0.3–0.4. Although every model showed significant correlation, some showed low correlation scores, such as 0.10 for the TELL task with PLS regression, due to the comparably large sample size for the statistical test (400–500). Therefore, we compared model performances with $R^2$ scores for better reliability. In terms of $R^2$, deep-learning-based models showed lower scores than other conventional methods. This result might be fueled by the limited data size, where millions of bits of data are usually used for neural network training to achieve better performances. For this reason, researchers using small-size datasets have frequently reported that conventional methods show promising results. In our case, the random forest model showed the best performance for all the tasks with $R^2$ scores over 0.13 and correlation coefficients over 0.38. We believe this result can be explained by the fact that tree-based models generally work well for interpolation setting, and our target scores had a limited range of 1–5 with several 100-ordered data samples.

In each component score level with random forest, although most results for the component skill scores showed a significant correlation, some components need further improvement. *Facial expression*, for instance, failed to estimate the scores for the *ASK* and *DECLINE* tasks. The differences between successful (*LISTEN, TELL*) and failed tasks (*ASK, DECLINE*) reflect the complexity of the facial expressions for each one. Since the discussion theme of successful tasks was simple, it did not flip from a positive to a negative topic. We set only positive themes for the agent utterances in the *LISTEN* task and asked users to share their positive feelings in the *TELL* task. Therefore, facial expressions could only be a neutral or a smiling face, an easy situation to measure. On the other hand, the failed tasks need both positive and negative facial expressions depending on the discussion phases. For example, the *ASK* task needs a contrite facial expression at the beginning. However, after the agent accepts the user’s request, the user can choose to express gratitude with a smile and continue to talk with an apologetic face. Since either expressions could be correct, depending on the dialogue context, measuring the appropriateness is technically challenging. One reason for this technical hurdle is that our current estimation system cannot accurately treat the synchronized timing of each multimodal feature since we averaged the features across all of the time frames of the session video. For such a timing-sensitive feature, we need to change the approach to sequential models to adequately capture its synchrony. Another challenge is capturing an appropriate representation of the speech contents. To deal with this problem, such cutting-edge NLP methods as speech-graph representations are required^45,46^.

In our additional ablation study on modalities, only the drop in visual modality led to a performance decrease by 0.12 for the TELL task and 0.10 for the ASK task in $R^2$. This indicates the importance of visual information for TELL and ASK. These results are intuitively understandable since these tasks require more expressive skills than LISTEN or DECLINE. Therefore, the model’s performance greatly decreased when such visual information as facial expressions and body gestures was reduced.

Regarding user preferences for the SST feedback, we found no significant group differences for any of the questions from Q1-1 to Q1-4 (Fig. 6). At the same time, however, note that several users commented on its usefulness for understanding how others objectively recognized their behaviors, implying that some users identified its practical usage for better improvements.

In the preference analysis on the feedback quantity, participants generally selected more feedback conditions (Table 7). However, the number of positive conditions of *“what feedback length do you prefer?”* was the only exception, where 18 people selected “1” and 16 selected “2”. Although the difference is relatively tiny, some people felt “2” positive feedback contents were as effective as one SST feedback. Based on this result, increasing the feedback content quantity (at least until it reaches two) and reducing the length (but keeping the same number of contents) of the language might improve the quality of the SST feedback. As additional analysis to confirm its appropriateness, we separately counted the amount of positive and corrective feedback. Interestingly, its average amounts for positive and corrective feedback were close to those selected by the participants with our human-agent SST system (2.09 and 2 for positive feedback, 0.59 and 1 for corrective feedback). Therefore, these values seem appropriate for SST feedback on both human–human and human-agent SSTs.

## Conclusion

We developed a social-skill-estimation model using audio-visual-text features with machine learning models. Despite the complex nature of social skills, we achieved reasonably high estimation performance with a 0.63 maximum Spearman’s correlation coefficient for the estimation models. Furthermore, we developed an automatic SST feedback system based on these estimated scores for an automated SST system under the supervision of clinically-experienced SST trainers. By conducting a user-study on the feedback, we investigated the best feedback conditions for recorded video. The preferred amount of positive/corrective feedback resulted in a combination of 2-positive and 1-corrective feedback. Our result suggests that recorded video is not a statistically effective component of an SST feedback system. With an additional investigation on human–human SST by experienced trainers, we confirmed the appropriateness of the best quantities of feedback by verifying that they nearly equaled the average feedback amount by professional SST trainers in a human–human setting (Supplementary Information).

In the next step of this study, we plan to investigate our system’s training effects in both short-term and long-term experiments. From a technical perspective, since we estimated the scores at the abstract level with random forest models, we believe that an estimation-feedback system with time-sequential modeling and visualization techniques can enhance the training performance of users. However, time-sequential labeling requires several annotators and labeling time, both of which are issues that we need to overcome. As another research possibility with the dataset, analysis of clinical group differences is an interesting direction. In this paper, we did not analyze the clinical group differences in communicative interaction since our focus was machine learning predictions and feedback analyses. However, a paper reports that ASDs and SZs can be differentiated with such disorder-specific positive symptoms (the presence of atypical behaviors) as hallucinations or positive formal thought disorder (e.g., disorganized thinking and incoherent speech) and are commonly seen in SZs^35^. Although these group comparisons are ongoing research areas, computational comparison of the clinical groups might give interesting insights for better treatments. Moreover, although technically challenging, personalizing SST feedback should be helpful for automated SST systems. As we mentioned in “Methods” section, for those with schizophrenia and who struggle with hallucinations, clinicians usually give short and clear feedback. Toward maximizing training effects, systems should have such a functionality in the future.

## Supplementary Information

List of supplemental materials.

Example video of human-agent social skills training.

## Data Availability

The dataset is available from Hiroki Tanaka (hiroki-tan@is.naist.jp) by e-mail upon reasonable request.
