# Supplementary material for: Automatic evaluation-feedback system for automated social skills training
Source: Sci Rep. 2023 Apr 26;13:6856. doi: 10.1038/s41598-023-33703-0 (PMC10133273; doi:10.1038/s41598-023-33703-0)
Supplement: Supplementary file 1 — List of supplemental materials. [file 41598_2023_33703_MOESM1_ESM.pdf]

Legend/caption of supplemental materials

Supplemental1:

- File name : Supplemental1_human-agent_SST_demo.mp4
- Caption : Demo video of our human-agent SST system
- Legend : N/A
